# Neoadjuvant FOLFOXIRI prior to chemoradiotherapy for high-risk (“ugly”) locally advanced rectal cancer: study protocol of a single-arm, multicentre, open-label, phase II trial (MEND-IT)

**DOI:** 10.1186/s12885-022-09947-w

**Published:** 2022-09-06

**Authors:** K. van den Berg, D. P. Schaap, E. L. K. Voogt, T. E. Buffart, H. M. W. Verheul, J. W. B. de Groot, C. Verhoef, J. Melenhorst, J. M. L. Roodhart, J. H. W. de Wilt, H. L. van Westreenen, A. G. J. Aalbers, M. van ‘t Veer, C. A. M. Marijnen, J. Vincent, L. H. J. Simkens, N. A. J. B. Peters, M. Berbée, I. M. Werter, P. Snaebjornsson, H. M. U. Peulen, I. G. van Lijnschoten, M. J. Roef, G. A. P. Nieuwenhuijzen, J. G. Bloemen, J. M. W. E. Willems, G. J. M. Creemers, J. Nederend, H. J. T. Rutten, J. W. A. Burger

**Affiliations:** 1 Department of Medical Oncology, Catharina Hospital, Eindhoven, the Netherlands; 2 Department of Surgery, Catharina Hospital, Eindhoven, the Netherlands; 3 Department of Gastrointestinal Oncology, Netherlands Cancer Institute, Amsterdam, the Netherlands; 4 Department of Medical Oncology, Amsterdam University Medical Centres, Amsterdam, the Netherlands; 5 Department of Medical Oncology, Radboud University Medical Centre, Nijmegen, the Netherlands; 6 Department of Medical Oncology, Isala Oncology Centre, Zwolle, the Netherlands; 7 Department of Surgical Oncology, Erasmus MC Cancer Institute, Rotterdam, the Netherlands; 8 Department of Surgery, Maastricht University Medical Centre, Maastricht, the Netherlands; 9 Department of Medical Oncology, University Medical Centre Utrecht, Utrecht, the Netherlands; 10 Department of Surgery, Radboud University Medical Centre, Nijmegen, the Netherlands; 11 Department of Surgery, Isala, Zwolle, the Netherlands; 12 Department of Surgical Oncology, Netherlands Cancer Institute, Amsterdam, the Netherlands; 13 Department of Research and Education, Catharina Hospital, Eindhoven, the Netherlands; 14 Department of Radiation Oncology, Netherlands Cancer Institute, Amsterdam, the Netherlands; 15 Department of Radiation Oncology, Leiden University Medical Center, Leiden, the Netherlands; 16 Department of Medical Oncology, Elkerliek Hospital, Helmond, the Netherlands; 17 Department of Medical Oncology, Maxima Medical Centre, Veldhoven, the Netherlands; 18 Department of Medical Oncology, St. Jans Hospital, Weert, the Netherlands; 19 Department of Radiation Oncology (MAASTRO), GROW School for Oncology and Reproduction, Maastricht University Medical Centre+, Maastricht, the Netherlands; 20 Department of Medical Oncology, Rijnstate Hospital, Arnhem, the Netherlands; 21 Department of Pathology, Netherlands Cancer Institute, Amsterdam, the Netherlands; 22 Department of Radiation Oncology, Catharina Hospital, Eindhoven, the Netherlands; 23 Department of Pathology, PAMM Laboratory for Pathology and Medical Microbiology, Eindhoven, the Netherlands; 24 Department of Nuclear Medicine, Catharina Hospital, Eindhoven, the Netherlands; 25 Department of Medical Oncology, Anna Hospital, Geldrop, the Netherlands; 26 Department of Radiology, Catharina Hospital, Eindhoven, the Netherlands; 27 GROW School for Oncology and Reproduction, Maastricht University, Maastricht, the Netherlands

**Keywords:** Locally advanced rectal cancer, Induction chemotherapy, Neoadjuvant chemotherapy, Neoadjuvant treatment, Pathological complete response, Clinical complete response, Complete response

## Abstract

**Background:**

The presence of mesorectal fascia (MRF) invasion, grade 4 extramural venous invasion (EMVI), tumour deposits (TD) or extensive or bilateral extramesorectal (lateral) lymph nodes (LLN) on MRI has been suggested to identify patients with indisputable, extensive locally advanced rectal cancer (LARC), at high risk of treatment failure. The aim of this study is to evaluate whether or not intensified chemotherapy prior to neoadjuvant chemoradiotherapy improves the complete response (CR) rate in these patients.

**Methods:**

This multicentre, single-arm, open-label, phase II trial will include 128 patients with non-metastatic high-risk LARC (hr-LARC), fit for triplet chemotherapy. To ensure a study population with indisputable, unfavourable prognostic characteristics, hr-LARC is defined as LARC with on baseline MRI at least one of the following characteristics; MRF invasion, EMVI grade 4, enlarged bilateral or extensive LLN at high risk of an incomplete resection, or TD. Exclusion criteria are the presence of a homozygous DPD deficiency, distant metastases, any chemotherapy within the past 6 months, previous radiotherapy within the pelvic area precluding standard chemoradiotherapy, and any contraindication for the planned treatment. All patients will be planned for six two-weekly cycles of FOLFOXIRI (5-fluorouracil, leucovorin, oxaliplatin and irinotecan) prior to chemoradiotherapy (25 × 2 Gy or 28 × 1.8 Gy with concomitant capecitabine). A resection will be performed following radiological confirmation of resectable disease after the completion of chemoradiotherapy. A watch and wait strategy is allowed in case of a clinical complete response. The primary endpoint is the CR rate, described as a pathological CR or a sustained clinical CR one year after chemoradiotherapy. The main secondary objectives are long-term oncological outcomes, radiological and pathological response, the number of resections with clear margins, treatment-related toxicity, perioperative complications, health-related costs, and quality of life.

**Discussion:**

This trial protocol describes the MEND-IT study. The MEND-IT study aims to evaluate the CR rate after intensified chemotherapy prior to concomitant chemoradiotherapy in a homogeneous group of patients with locally advanced rectal cancer and indisputably unfavourable characteristics, defined as hr-LARC, in order to improve their prognosis.

**Trial registration:**

Clinicaltrials.gov: NCT04838496, registered on 02–04-2021 Netherlands Trial Register: NL9790.

**Protocol version:**

Version 3 dd 11–4-2022.

## Background

Locally advanced rectal cancer (LARC) is often defined as a clinically staged (c) T3 tumour within 1 mm from the mesorectal fascia (MRF), a cT4 tumour, cN2 disease, or rectal cancer in the presence of extramesorectal lymph nodes [1–3]. Standard treatment consists of neoadjuvant chemoradiotherapy prior to surgery, i.e. a total mesorectal excision [1, 4, 5]. However, despite optimal treatment, LARC is associated with local recurrence and distant metastasis rates ranging between 5–10% and 25–40%, respectively [5–12]. This has resulted in an ongoing search for better treatment regimens in patients at high risk of treatment failure.

The main aim of neoadjuvant treatment is to obtain downstaging to facilitate a resection with clear resection margins (R0) [5, 6, 13]. An R0 resection is an important prognostic factor for disease-free survival, particularly due to an improved local recurrence-free survival [5, 8, 14, 15]. In addition, the introduction of neoadjuvant treatment regimens has translated into new treatment strategies, focusing on organ preservation [16–19]. Previous studies reported a pathological complete response (pCR) rate, i.e. the absence of malignant cells on pathological examination after surgery, in over 15% of the patients treated with neoadjuvant chemoradiotherapy [16, 20–22]. Improved long-term outcomes, probably related to a favourable tumour biology, have been suggested in patients with a pCR [22, 23]. Subsequently, an organ preservation strategy with active surveillance, referred to as a watch and wait (W&W) strategy, has been introduced to prevent unnecessary surgical morbidity and mortality without comprising (disease-free) survival [16–19].

Nonetheless, substantial improvements in distant metastasis rates and overall survival for patients with LARC are lagging behind [3]. The administration of adjuvant chemotherapy to reduce distant metastasis rates and improve overall survival in rectal cancer is controversial [3, 24, 25]. As an alternative, the addition of neoadjuvant chemotherapy to the current treatment regimen has been suggested [26–29]. A matched case–control study observed an improved complete response (CR) rate of 28% compared to 9% (p = 0.013) with the addition of doublet chemotherapy to neoadjuvant treatment in patients with unfavourable LARC, defined as any cT4 rectal cancer, or cT2/3 rectal cancer with extramural venous invasion (EMVI), and/or tumour deposits, and/or cN2 disease on magnetic resonance imaging (MRI) [30]. These results on CR are in line with those of three recently published, phase III randomised controlled trials in LARC [28, 31, 32]. In the PRODIGE 23 trial patients with cT3 rectal cancer eligible for chemoradiotherapy and cT4 rectal cancer were randomly assigned to either chemoradiotherapy alone or neoadjuvant FOLFIRINOX (fluorouracil, irinotecan, leucovorin, oxaliplatin) followed by chemoradiotherapy prior to surgery [32]. The RAPIDO trial included patients with an involved MRF (the primary tumour or a lymph node on a distance of ≤ 1 mm from the MRF), cT4 disease, extramural vascular invasion, cN2 disease, or enlarged lateral lymph nodes [33]. These patients were treated with either chemoradiotherapy alone or short-course radiotherapy followed by neoadjuvant doublet chemotherapy (a fluoropyrimidine in combination with oxaliplatin; CAPOX/FOLFOX) [33]. Despite the improvement in CR rate and 3-year disease-free survival / disease-related treatment failure in patients treated with neoadjuvant chemotherapy, no substantial improvements in overall survival were reported in either trial [32, 33]. Moreover, in the RAPIDO trial, the locoregional failure rate at 5 years was higher in the experimental group compared to the standard of care group (10% versus 7%, *p* = 0.038), whereas the significant difference in distant metastasis rate (23% versus 31%, *p* = 0.011) remained favourable for the experimental group [34]. In the STELLAR trial, patients with cT3-4 and/or node-positive disease were randomised between either neoadjuvant chemoradiotherapy or short-course radiotherapy followed by doublet chemotherapy [28]. A non-inferior 3-year disease-free survival was observed in the experimental arm, and preliminary results suggested a benefit in 3-year overall survival in this group [28]. However, a longer follow-up is awaited to draw definite conclusions, considering the disappearance of an overall survival benefit in the Polish II trial after a 8 year follow-up period [35]. Apart from these survival outcomes, an increase in preoperative ≥ grade 3 adverse events was reported in the experimental arms [28, 32, 33]. Nevertheless, the administration of both doublet and triplet chemotherapy did not result in significantly less patients proceeding to surgery compared to the standard treatment arms [32, 33].

Hence, neoadjuvant chemotherapy in combination with (chemo)radiotherapy may improve short- and long-term outcomes in LARC. However, the absence of a substantial improvement in overall survival, the increased risk of treatment-related toxicity, possibly resulting in treatment delays, the differences in patient selection and treatment regimens among different studies, and the underrepresentation of patients with the prognostic most unfavourable rectal tumours hamper the clinical implementation of neoadjuvant chemotherapy in rectal cancer [29, 31, 36–40]. Moreover, overrepresentation of patients who do not need additional systemic treatment for cure, may also dilute positive oncological results. As a consequence, it has been hypothesised that total neoadjuvant treatment, consisting of both neoadjuvant chemotherapy and chemoradiotherapy, should be preserved for a select group of patients facing the worst prognosis [31].

Since the start of the RAPIDO trial in 2011, imaging-based staging has been further refined [33]. High-risk imaging-based features were more routinely described and studied, which resulted in the recognition of clinically important, unfavourable characteristics on MRI [33, 41–43]. An involved MRF, defined as a distance of ≤ 1 mm to the MRF, has already been recognized as an important, unfavourable prognostic feature for several years [44, 45]. In addition, the identification of EMVI, tumour deposits, or enlarged extramesorectal (lateral) lymph nodes (LLN) with a short-axis of at least 7 mm on baseline MRI has been suggested to identify patients with the most ‘ugly’ LARC, at high risk of failure on current treatment regimens [33, 42, 46–51]. A significantly decreased disease-free and overall survival rate has been described for patients with EMVI-positive rectal cancer, with 5-year distant metastasis rates up to 45.2%, compared to EMVI-negative rectal cancer (25.7%) [12]. In addition, the presence of tumour deposits on baseline MRI has been identified as an independent prognostic factor, indicating a poor survival [42, 52]. Lastly, a lateral local recurrence rate of up to 19.5% has been described in patients with lateral lymph nodes, located in the iliac or obturator compartment, with a short-axis of at least 7 mm on baseline MRI despite treatment with chemoradiotherapy prior to a total mesorectal excision [48].

Hence, these imaging-based, high-risk features may be used to identify patients with LARC facing the worst prognosis, at high risk of failing on current treatment regimens. Nevertheless, it has been described that the recognition of imaging-based MRF involvement might be overestimated [45, 51, 53, 54]. In addition, the presence of EMVI is evaluated based on a scoring system, classifying both grade 3 and 4, in contrast to grade 0–2, as EMVI-positive [55]. Grade 3 might comprise more subtle, possibly debatable, EMVI, whereas grade 4 describes evident EMVI with obvious vessel abnormalities [55]. Therefore, we hypothesise that the presence of evident MRF invasion, EMVI grade 4, extensive or bilateral enlarged lateral lymph nodes, or tumour deposits, we refer to as MEND criteria, will correctly identify a homogeneous group of patients with high-risk (hr-) LARC, facing the worst prognosis.

This select group of patients fulfilling the MEND criteria might benefit from an intensified treatment regimen. However, these patients were underrepresent in previous trials [28, 32, 33]. The addition of intensified induction chemotherapy (IT), consisting of FOLFOXIRI (5-fluorouracil, irinotecan, leucovorin, oxaliplatin) has been suggested. This triplet chemotherapy regimen has been associated with a significantly improved tumour response of over 10%, progression-free survival and overall survival compared to a doublet chemotherapy regimen in patients diagnosed with metastatic colorectal cancer [56–58]. The PRODIGE 23 trial confirmed that the majority of the patients in the experimental arm was treated with chemoradiotherapy after neoadjuvant, triplet chemotherapy (95%), compared to 99% in the standard of care arm (*p* = 0.019) [32]. Moreover, the addition of both doublet and triplet chemotherapy to neoadjuvant (chemo)radiotherapy did not result in less patients proceeding to surgery compared to standard treatment [32, 33].

In conclusion, personalised medicine in rectal cancer is warranted to improve surgical and oncological outcomes and to prevent both over- and undertreatment. The primary aim of this single-arm, phase II study is to evaluate whether neoadjuvant chemotherapy with FOLFOXIRI prior to chemoradiotherapy provides an increased CR rate in a homogeneous group of patients with hr-LARC, fulfilling the MEND criteria, compared to current literature.

## Methods/design

### Aim, design, and study setting

This is a multicentre, single-arm, open-label, phase II trial. All included patients will be treated with induction chemotherapy, consisting of FOLFOXIRI, prior to chemoradiotherapy. The study is registered in Clinicaltrials.gov (NCT04838496) and in the Netherlands Trial Register (NL9790), where a list of participating centres can be obtained. The primary aim of this study is to evaluate whether the addition of neoadjuvant chemotherapy with FOLFOXIRI prior to chemoradiotherapy results in a higher pCR rate or sustained clinical complete response (cCR) rate at 1 year in patients with hr-LARC, facing a particularly poor prognosis.

### Eligibility criteria

Patients, at least 18 years of age, with histopathologically confirmed, (deemed) resectable hr-LARC, with the low border of the tumour at or below the sigmoidal take-off as established on MRI, with a World Health Organization (WHO) performance status of ≤ 1, and fit for (modified dose) triplet chemotherapy are eligible for inclusion [59, 60]. An expected gross incomplete resection with overt tumour remaining in the patient after resection, tumour invasion in the neuroforamina, encasement of the ischiadic nerve and invasion of the cortex from S2 upwards are considered unresectable. Patients will be excluded in case of metastatic disease at inclusion, a homozygous DPD (dihydropyrimidine dehydrogenase) deficiency, any chemotherapy within the past 6 months, prior radiotherapy in the pelvic area interfering with the planned study treatment, any contraindication for the planned treatment, or concurrent malignancies that interfere with the planned study treatment or prognosis. Enlarged iliac or inguinal lymph nodes and aspecific lung nodules do not result in exclusion. All patients will be discussed during a multidisciplinary team (MDT) meeting to ensure their eligibility.

### Definition of hr-LARC

To ensure a study population with the most unfavourable prognosis in terms of disease-free survival, the definition of hr-LARC is based on the previously described, imaging-based characteristics that represent LARC at particularly high risk of treatment failure. Hr-LARC is therefore defined as LARC with on baseline MRI at least one of the following characteristics; indisputable tumour invasion into the MRF, EMVI grade 4, bilateral LLN with a SA of ≥ 7 mm, or extensive LLN involving pelvic side wall structures such as vessels, nerves or the ureter, at high risk of treatment failure, or tumour deposits. The presence of an “involved” or “threatened” MRF, or EMVI grade 3 is insufficient for inclusion in this study [55]. Moreover, consensus should be reached about the eligibility during a MDT meeting. A syllabus containing information and images of eligible hr-LARC will be provided to all participating hospitals.

### Interventions

**Fig. 1:**
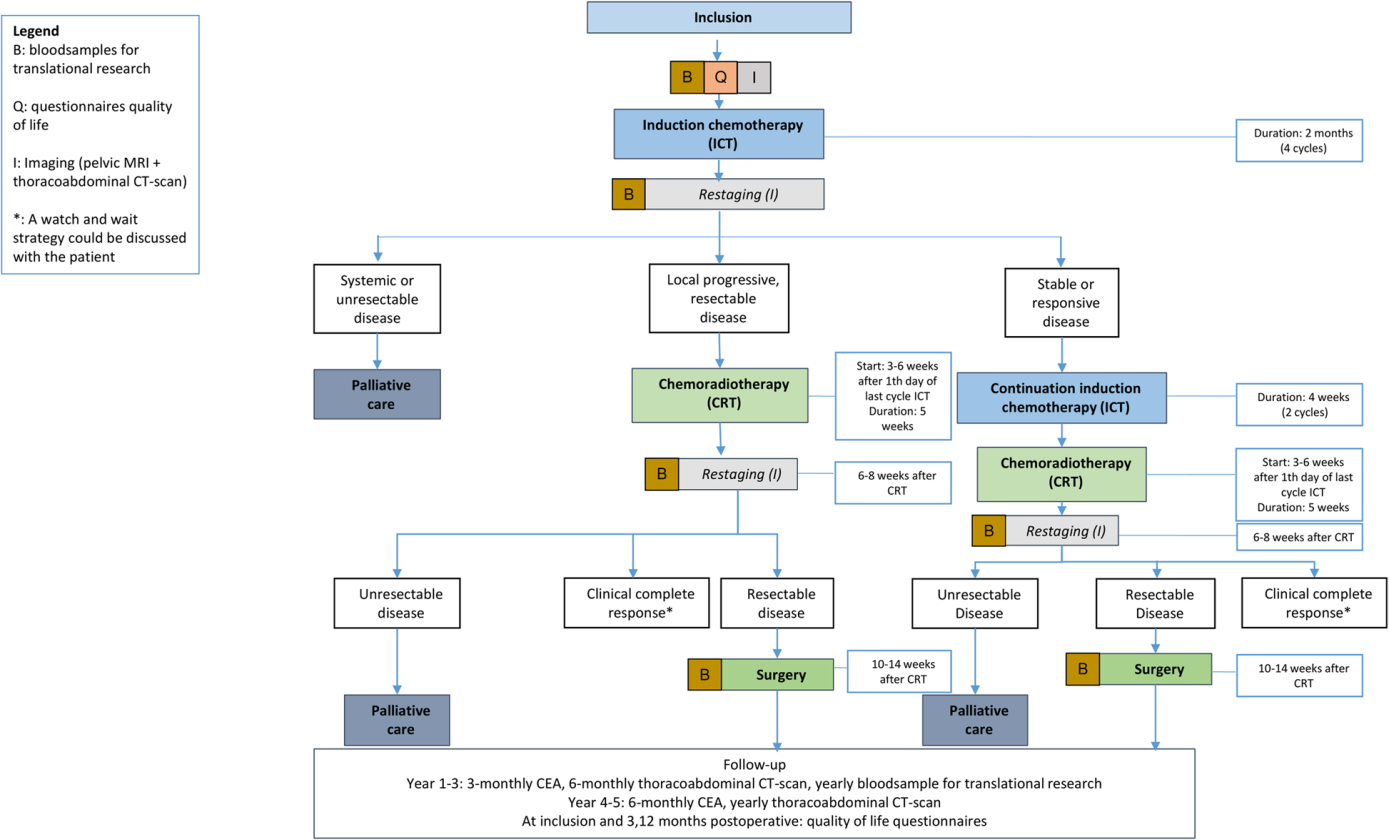
Study flow diagram

Figure 1 provides a general flowchart of the study.

#### Neoadjuvant chemotherapy

All included patients will receive neoadjuvant (induction) chemotherapy within 4 weeks after inclusion. Neoadjuvant chemotherapy consists of six two-weekly cycles of FOLFOXIRI (irinotecan 165 mg/m^2^ Body Surface Area (BSA), oxaliplatin 85/m^2^ BSA, leucovorin 400 mg/m^2^ BSA, 5-flourouracil 3200 mg/m^2^ BSA). Dose reductions are permitted on discretion of the medical oncologist.

Restaging will be performed with a pelvic MRI and a thoraco-abdominal computed tomography (CT) scan after four cycles of FOLFOXIRI. The timing of restaging should not interfere with the potential continuation of neoadjuvant chemotherapy. Radiological evaluation will be performed by a radiologist with expertise in abdominal imaging conform a standard operating procedure. The results will be discussed during a MDT meeting in one of the participating centres in the attendance of at least a surgical oncologist, a medical oncologist, a radiation oncologist, and a radiologist with expertise in abdominal imaging. If a patient has stable or responsive disease, neoadjuvant chemotherapy will be continued with two additional cycles of FOLFOXIRI. Subsequently, chemoradiotherapy will start within 3–6 weeks after the last cycle of neoadjuvant chemotherapy. If progressive, but still resectable disease is assumed during restaging, no further systemic therapy will be administered, and the patient will start with chemoradiotherapy within 3–6 weeks. If restaging suggests progressive, unresectable disease or the presence of distant metastases, the best palliative treatment will be offered according to standard of care.

#### Chemoradiotherapy

After neoadjuvant chemotherapy, all patients with (deemed) resectable disease will receive concomitant chemoradiotherapy according to standard of care within 3–6 weeks after the first day of the last cycle of FOLFOXIRI. Chemoradiotherapy will consist of 50 Gy or 50.4 Gy delivered in 25 or 28 fractions, respectively, with concomitant capecitabine (825 mg/m2 BSA) twice daily on radiotherapy days.

#### Treatment evaluation

Six to eight weeks after the last day of the chemoradiotherapy, restaging will be performed with a pelvic MRI and a thoraco-abdominal CT scan. The results will be discussed during a MDT meeting. A surgical resection will be planned for all patients with resectable disease after neoadjuvant treatment. In case of a cCR, a W&W strategy with close surveillance may be discussed with the patient by the treating physician according to standard of care. The presence of distant metastases or unresectable local disease will result in the best palliative treatment according to standard of care.

#### Surgery

Surgery will be performed according to the standard of care by a surgical team with experience in rectal cancer surgery, 10–14 weeks after the completion of chemoradiotherapy. The type and extent of the surgery and the possible addition of intraoperative radiotherapy (IORT) will be left to the discretion of the surgeon.

#### Follow-up

Patients’ follow-up will be performed according to standard of care every 3 months in the first 3 years and every 6 months thereafter up to 5 years postoperatively [1]. The level of carcinoembryonic antigen (CEA) will be determined at every follow-up moment. If during the follow-up the CEA level increases, a thoraco-abdominal CT scan will be performed according to the Dutch guidelines. At 6, 12, 18, 24, 30, 36, 48- and 60-months post-operatively, a thoraco-abdominal CT scan will be performed. A FDG-PET/CT scan is allowed, but not mandatory. In case of a W&W approach, the aforementioned follow-up will be expanded according to Dutch standard of care [23].

#### Questionnaires

All patients participating in the study will receive validated quality of life questionnaires after informed consent. Patients will be asked to complete the EORTC QLQ-C30, QLQ-CR29 (from the European Organisation for Research and Treatment of Cancer Quality of Life Group), and EQ-5D-5L (from the EuroQoL Group, Rotterdam, the Netherlands) at inclusion, 3, and 12 months post-operatively [61–63]. Patients receive questionnaires either by email or on paper, according to their own preferences.

#### Translational research

All patients will be asked for informed consent to collect blood samples for future translational research. An additional 20 ml blood is drawn during regular blood draws before start of the neoadjuvant chemotherapy, before chemoradiotherapy, before surgery, three months after surgery, and once a year during three years of follow-up, resulting in 7 samples. In addition, all patients will be asked for informed consent to collect fresh frozen tumour tissue obtained during surgery for translational research.

### Outcomes

The primary outcome of this study is the CR rate, defined as the presence of a pCR or a cCR. A pCR is defined as the absence of viable tumour cells at pathological examination of the resected specimen [22, 64]. A cCR is defined as the sustained absence of tumour tissue 1 year after treatment as assessed during clinical evaluations [16, 17]. Since the MEND-IT study is a phase II study, the CR rate observed in this trial will be compared to the CR rate as described in current literature for patients with hr-LARC treated with chemoradiotherapy alone (i.e. 10%) [32, 33]. In addition, the results of the MEND-IT trial will be compared to results of comparable cohorts treated with different treatment regimens.

Secondary objectives comprise the number of patients proceeding to surgery, the 3- and 5-year local recurrence-free, distant metastasis-free, progression-free, disease-free and overall survival. In addition, radiological response, pathological response, R0 resection rate, toxicity, treatment compliance rate, surgical morbidity, quality of life, and costs will be evaluated. Local recurrence-free survival is defined as the interval between surgery and a local recurrence. Metastasis-free survival is defined as the interval between inclusion and the detection of distant metastases. Progression-free survival will be calculated from inclusion onwards to the date of clinically or histopathologically proven distant metastases, a local recurrence, or the date of death from any cause, whichever occurs first. Disease-free survival will be calculated from the date of surgery onwards or the date of the second restaging MRI in case of a cCR until the date of a local recurrence, distant metastasis, or death from any cause, whichever occurs first. Overall survival will be calculated from the date of inclusion until the date of death from any cause. Toxicity from neoadjuvant treatment will be presented according to the NCI (US National Cancer Institute) Common Terminology Criteria for Adverse Events (CTCAE) v5.0 [65]. Perioperative complications will be presented according to the Clavien-Dindo grading system [66]. Pathological response will be presented according to the Mandard grading system [64].

### Sample size

A 10% CR rate is assumed in patients diagnosed with hr-LARC, treated with chemoradiotherapy alone prior to surgery [30, 32]. Based on the available literature and a retrospective analysis of patients from our own institution a CR rate of 20% is hypothesised after neoadjuvant chemotherapy and chemoradiotherapy for hr-LARC [30, 32, 33]. A 5% significance level and a 90% power, resulted in a total of 121 required patients. A drop-out of 5% is expected, resulting in a total sample size of 128 patients.

### Recruitment

All eligible patients with hr-LARC presenting in one of the study centres will be identified by their physician and reviewed for eligibility during a MDT meeting.

### Data collection and management

Central data management will be performed by the research team of the Catharina Hospital Eindhoven. Local data management will be performed by the coordinating investigator or an in-hospital qualified local data management team. Data will be collected in a central study database with an electronic case report form (eCRF) according to the Good Clinical Practice guidelines and the Dutch legal requirements. Major protocol violations will be recorded.

### Statistical methods

Demographics, patient, and tumour characteristics will be presented for all patients. Continuous data will be reported as mean with a standard deviation or as median with an interquartile range or 95% confidence interval, depending on the distribution. Categorical data will be reported as count, including a percentage. All statistical tests will be two-sided and a P-value of less than 0.05 will be considered as statistically significant.

The proportion of patients with a CR, defined as a pCR or a sustained cCR, will be compared to the CR rate of a comparable group of patients treated with chemoradiotherapy alone before surgery, as described in the available literature (i.e. 10%), performing a chi-squared goodness-of-fit-test.

The Kaplan–Meier method will be used to display survival curves. In addition, Hazard Ratio’s will be calculated using the Cox proportional hazard regression model and will be accompanied by 95% confidence intervals. Perioperative morbidity, surgical characteristics, and histopathological findings will be presented for all patients treated with a surgical resection. The incidence of toxicity related to neoadjuvant treatment will be presented separately for neoadjuvant chemotherapy and chemoradiotherapy. Health-related quality of life is graphically presented across all time points. Moreover, quality of life will be compared during those different time points using the ‘repeated measures ANOVA’. Incremental costs are calculated for the extra costs per additional patient alive and the extra costs per additional quality adjusted life year, respectively. The 95% confidence intervals for (differences in) costs and health outcomes will be generated by non-parametric bootstrapping, drawing samples of the same size as the original samples.

### Data monitoring

A data safety monitoring board (DSMB) has been appointed to monitor patients’ safety and to advice the study steering committee on the continuation of the study after the interim analysis. The interim analysis will be performed when 50 patients have undergone surgery or entered a W&W approach. The number of patients not able to complete chemoradiotherapy and the number of patients with major postoperative morbidity (i.e. Clavien-Dindo ≥ 3) will be analysed [66]. Results will be discussed during a meeting of the DSMB, subsequently resulting in a recommendation about the continuation of the study. A premature termination of the study may be advised if more than 35% of the patients is unable to complete chemoradiotherapy due to treatment-related toxicity, or if more than 50% of the patients experience severe postoperative complications (i.e. Clavien-Dindo ≥ 3).

### Harms

All serious adverse events (SAEs) and/or suspected unexpected serious adverse reactions (SUSARs) will be reported to the coordinating investigator within 24 h after detection of the SAE. SAEs will be reported by the coordinating investigator to the accredited medical research ethics committee that approved the MEND-IT study protocol, using the web portal ToetsingOnline (https://toetsingonline.nl).

### Auditing

The study will be monitored by a qualified monitor from the Netherlands Comprehensive Cancer Organization (IKNL) based on a predetermined monitoring plan.

### Protocol amendments

All substantial protocol amendments will be presented to the competent authority, the medical research ethics committee, the institutional review boards of all study centres, the (principal) investigators and the trial registers.

### Confidentiality

Individual patient information obtained as a result of this study will be handled conform the Dutch guidelines regarding General Data Protection Regulation (in Dutch: AVG). Moreover, the use of study numbers will ensure patients’ confidentiality.

### Ancillary and post-trial care

There is no provision for ancillary or post-trial care in the MEND-IT study.

### Dissemination policy

The results of this study will be published in an international peer-reviewed journal. Moreover, results will be presented by offering an abstract to (inter)national congresses. Any presentation, publication, or abstract based on the results of this study must be approved by the trial steering committee and coordinating investigator.

## Discussion

Despite encouraging results of neoadjuvant chemotherapy in patients diagnosed with LARC, the clinical applicability remains a topic of debate due to varying inclusion criteria, varying treatment strategies, and the lack of improvement in overall survival in previous studies [31]. The addition of (intensified) neoadjuvant chemotherapy to the current treatment regimen may improve surgical and oncological outcomes in some patients, but may also prove unfeasible in many patients and even induce unnecessary risks [32, 33]. This highlights the need for uniform guidelines regarding patient selection and treatment regimens in LARC [26, 27, 31]. The aim of this multicentre, single-arm phase II study is to evaluate the CR rate of intensified neoadjuvant treatment, consisting of triplet chemotherapy (FOLFOXIRI) prior to chemoradiotherapy, in a select, homogeneous group of rectal cancer patients meeting the MEND criteria, and who are therefore facing the worst prognosis. We refer to this subgroup of locally advanced rectal cancer as “hr-LARC”. 

Triplet chemotherapy has been shown to be feasible and effective in patients with rectal cancer [32, 56]. Moreover, an improved tumour response has been described with FOLFOXIRI (triplet) chemotherapy compared to doublet chemotherapy in metastatic colorectal cancer [56–58]. The PRODIGE 23 trial observed an enhanced CR rate and 3-year DFS for patients treated with neoadjuvant triplet chemotherapy, consisting of FOLFIRINOX, prior to standard treatment with chemoradiotherapy and surgery [32]. Despite the intensity of triplet chemotherapy with the occurrence of serious adverse events in 20% of the patients, the vast majority of the patients in the experimental arm was able to proceed to chemoradiotherapy (95%) and surgery (92%) afterwards [32]. Nevertheless, patients with LARC facing the worst prognosis were underrepresented. We hypothesised that patients with hr-LARC, meeting the MEND criteria, may benefit most from intensive treatment in an early stage of the disease. Hence, the addition of triplet chemotherapy consisting of FOLFOXIRI to current standard treatment has been suggested.

With regard to this trial protocol, it could be questioned if chemotherapy should be administered after instead of prior to chemoradiotherapy given the beneficial results on CR rate and organ preservation in the OPRA and the CAO/ARO/AIO-12 trials [19, 31, 67]. However, no difference in DFS was reported in these trials. Definite recommendations for clinical practice regarding the timing of neoadjuvant chemotherapy are still awaited. Therefore, it has been suggested that the optimal timing of neoadjuvant chemotherapy might differ among patients and treatment strategies, highlighting the need for personalised treatment regimens [31, 67]. Considering the increased intensity of triplet chemotherapy compared to doublet chemotherapy, the administration of FOLFOXIRI prior to chemoradiotherapy will offer participants sufficient time to optimise their fitness ahead of surgery. Moreover, an improved effect of IORT, which is considered standard of care for many of these patients in the Netherlands, has been described with a limited time-interval between preoperative (chemo)radiotherapy and IORT [68, 69]. In addition, patients with hr-LARC have a particularly high risk of developing distant metastases. It has been hypothesised that the early eradication of micrometastatic disease by systemic therapy might contribute to a decreased distant metastasis rate [32].

The primary endpoint, i.e. CR rate, will evaluate treatment response in this select, relatively rare group of patients with hr-LARC. To observe a substantial improvement in long-term outcomes, i.e. disease-free and overall survival, larger study populations are required. Considering the intensity of treatment, the evaluation of a clinically relevant, treatment-related effect, i.e. CR rate in a smaller cohort, seems justified. Additionally, long-term outcomes will be evaluated. Moreover, extending the current phase II to a phase III trial could be considered based on the results. Therefore, results from this study will contribute to the personalisation of rectal cancer treatment and the development of an adequate and uniform treatment strategy for patients with hr-LARC.

## Data Availability

Access to the final trial dataset will be preserved for the central data manager, study statistician, coordinating investigator, and trial steering committee. No contractual agreements have been drawn limiting the access to data for these investigators. The dataset supporting the conclusions of this trial will be available on reasonable request.

## Abbreviations

AE: Adverse Event
AR: Adverse Reaction
c: Clinically staged
CR: Complete response
cCR: Clinical complete response
CEA: Carcinoembryonic antigen
CR: Complete response (pCR and cCR)
CRM: Circumferential resection margin
(PET) CT: (Positron emission tomography) computed tomography
DPD: Dihydropyrimidine dehydrogenase
DSMB: Data Safety Monitoring Board
eCRF: Electronic case report form
EMVI: Extramural venous invasion
FOLFOXIRI: 5-Fluorouracil, leucovorin, oxaliplatin, irinotecan
GDPR: General Data Protection Regulation;
Gy: Gray
hr-LARC: High-risk locally advanced rectal cancer
IORT: Intraoperative radiotherapy
IV: Intravenously
LARC: Locally advanced rectal cancer
LLN: Lateral lymph nodes
MDT: Multidisciplinary team
METC: Medical research ethics committee (MREC); in Dutch: medisch-ethische toetsingscommissie (METC)
MRF: Mesorectal fascia
MRF +: Mesorectal fascia invasion
MRI: Magnetic resonance imaging
R0 resection: A resection with clear resection margins
pCR: Pathological complete response
SA: Short-axis
(S)AE: (Serious) Adverse Event
SUSAR: Suspected unexpected serious adverse reaction
TD: Tumour deposits
WHO: World Health Organization
WMO: Medical Research Involving Human Subjects Act; in Dutch: Wet Medisch-wetenschappelijk Onderzoek met Mensen
W&W: Watch and wait
